# Small Things, Micro-Affirmations and Helpful Professionals Everyday Recovery-Orientated Practices According to Persons with Mental Health Problems

**DOI:** 10.1007/s10597-018-0245-9

**Published:** 2018-02-08

**Authors:** Alain Topor, Tore Dag Bøe, Inger Beate Larsen

**Affiliations:** 1 Department of Psychosocial Health, University of Agder, Kristiansand, Norway; 2 Department of Social work, Stockholm University, Stockholm, Sweden

**Keywords:** Recovery, Professional practice, Helpful relationships, Micro affirmations, Small things

## Abstract

The aim of this study is to present concrete descriptions of the content in the construction of helpful relationships with staff, according to users. Starting with the re-occurring concept of the meaning of “little things” in recovery studies, a literature review was done. A thematic analysis shows that small things play an important role in improving a person’s sense of self. Small things seem to be an invisible but effective parts of a recovery-orientated practice, but they might be defined as unprofessional and their efficacy negated.

## Background

In studies about helping factors in persons’ recovery process, it is possible to notice the occurrence of anecdotes about how seemingly casual events can have an important impact on a person´s well-being and development (Davidson and Strauss 1992).

Small things have been noticed in studies about stigma and described as “micro-aggressions” (Sue 2010; Gonzalez et al. 2015). In this paper we will focus on small things having a positive role for a person’s recovery process, known as micro-affirmations. There is a copious amount of literature showing that most psychotherapeutic methods seem to be as effective as each other. Thus, their efficacy cannot primarily be explained by their specific technical components, but depend on something else, common to the different effective practices (Frank and Frank 1991; Wampold and Imel 2015). Lately, studies have focused on common factors in treatment as usual and about the recovery process for persons with severe mental health problems (McCabe and Priebe 2004; Davidson and Chan 2014).

Recovery has been described primarily as personal journey (Anthony 1993). It has been pointed out that a risk with this focus on the person is considering the user out of his/her social context and putting the burden of recovery solely on the person (Rose 2014; Price-Robertson et al. 2016; Topor et al. 2011).

Social aspects of recovery were considered in early studies (Breier and Strauss 1984, Warner 2004), and a renewed interest in analyzing recovery as a social process has been noticed during the last decade (Davidson et al. 2006; Borg and Davidson 2007; Tew et al. 2012). An important aspect of these studies is their focus on social relationships (Topor 2001; Borg and Davidson 2007) and helpful relationships with professionals in different settings (Denhov and Topor 2012; Borg and Kristiansen 2004; Topor and Denhov 2015). These studies are mostly based on users’ experience-based knowledge and they emphasize the importance of being heard, seen and respected. Other notions commonly mentioned are empathy, interest and engagement (Borg and Kristiansen 2004; Topor 2001).

A recurring phenomenon in these studies is the special role of “small things”. These “small things” are not part of a treatment procedure; they are not meant to have an impact on the person’s problems nor to contribute to his/her recovery process. This paradoxical description constitutes the point of departure for the present study. Small things can be found in recovery studies in two contexts. The first is in everyday life, as in Davidson et al. (2006, p 157): “This sense of mastery can come from seemingly trivial experiences of being able to turn on and off one’s radio”. The second is part of the interaction between the person and one professional. Despite the importance they are given in recovery processes, we lack an overview about what these small things are and how they work.

In this paper we will focus on the interaction between persons experiencing severe mental health problems and staff members employed to care for and support them. We aim to study how these small things are described and how those with a personal experience describe their impact and contribution to their recovery journey. Finally, we will look at how small things influence our understanding of professionality in the mental health field.

## Method

To get an overview of the occurrence of small things, we started with a search in Google scholar and Psychinfo, combining words such as ‘small things’ and ‘mental health’ and/or ‘recovery’ and/or ‘helpful relation*’. These searches were not conclusive as they mostly resulted in references to “small things” persons with mental health problems could do to help themselves.

Instead, we gathered articles we knew about that had been published in peer-reviewed journals. We asked colleagues for their help to find work about “small things”. We also read the reference lists in the actual literature.

The analysis presented is based on 26 articles, three contributions in books, and two PhD theses, and has no pretention to be exhaustive.

Studies about helping relationships might mention phenomena that were described as little things in the literature we reviewed, but without denominating them as small things. We will refer to some of those studies only to show the diffusion of the term ‘small things’. An example is Green et al. (2008) where the authors list “call returning during off-hours, spending extra time when it was needed” (p. 9), “Listening, understanding, believing and knowing the patient” and “Friendship and mutuality” (p. 10), but do not categorize their findings as small things.

We used thematic analysis according to the work of Braun and Clarke (2006) because we found this method could be adapted to our aim of identifying, analyzing and reporting themes related to small things. Thematic analysis could be used to analyze various aspects of the phenomenon described as helpful by the persons themselves.

The first step in the analytical process was to become familiar with the data, so we read the collected texts several times. Then we generated initial codes and noted the parts mentioning “tiny” phenomena connected to professionals and being of help to the person. The third step was to develop the themes from the collected codes. After that we reviewed the themes and named them, looking for illustrative excerpts. The analysis resulted in six themes about small things, which we named: (a) appellations, (b) constitution, (c) forms, (d) functions, (e) consequences for the person (f) consequences for the professional and professionality and (g) consequences for practice.

## Findings

The presentation of our findings is divided according to the themes.

### Appellations

The concept of small things occurs in different articles and contributions to books (Davidson and Johnson 2013; Ness 2016; Schranck et al. 2011; Topor 2001; 2004, 2014; Ware et al. 2004), but is not the only appellation related to the phenomena we are looking at.

Rowe (2008), starting from research about micro-aggressions (Deegan 2004; Gonzales et al. 2005), coined the concept of “micro-affirmations”, which she defined as:

“Apparently small acts, which are often ephemeral, hard-to-see, events that are public and private, often unconscious but very effective, which occur wherever people wish to help other to succeed” (46).

Coining the concept “micro-affirmations” points to the ethical aspect that seems to be present in most appellations: what is said or done involve the experience of being valued by another in some way (Ness 2016; Bøe et al. 2015).

The size aspect of the phenomena seems present in most appellations, such as in “small gestures” (Klevan et al. 2017; Andersson 2016), “apparently small acts” (Borg and Kristiansen 2004), “… simple or small things” (Schrank et al. 2011), “little issues” (Rowe 2008), “little extras” (Topor 2014) “subtle cues” and “micro-gestures” (Costin 2016) “something as apparently trivial” (Davidson and Strauss 1992). The negligible aspect of the phenomena can also be noticed in “simple gestures” (Nodeland et al. 2016). The tininess of the things is often simultaneously problematized with words like “apparently” (Davidson and Johnson 2013). Their outsider position in relation to what really counts is stressed by the use of words like “extra-ness” (Ware et al. 2004).

Something else that is present in most appellations of small things is the relational aspect, most easily seen in the Scandinavian languages as the presence of a “good chemical reaction” (Ness 2016, p. 59), “good chemistry” (Borg and Kristiansen 2004, p. 500) and “personal chemistry” (Denhov and Topor 2012) between two persons.

Thus the common features of these phenomena are that they are tiny and that despite their tininess they might play an important role in improving a person’s state and situation. “These gestures, no matter how insignificant they might seem, carry infinite weight in a person’s sense of wellness and recovery” (Costin 2016, p. 122). This is also stressed in some of the articles’ titles as “small things of great importance” (Topor 1999; Nodeland et al. 2016).

### Constitution

What make something a “small thing”?

#### Context

A common feature seems to be that small things are characterized by their everydayness (Skatvedt 2017; Ljungberg et al. 2015; Nodeland et al. 2016; Davidson and Johnson 2013). What makes them important is that they occur in situation contrasting everyday life relations and situations. Thus, what makes a thing “small” seems to be the context in which it appears. Small things are small in relation to other things, i.e. big things.

In contexts where people with different diagnoses are treated, “big things” are the treatment and the maintenance of professional distance. Adequate interventions and the person’s adherence to the treatment are keys to improvement. Small things are situations without “formally declared therapeutic value” (Skatvedt 2017, p. 5).

#### In Contrast to the Professional Role

The role of the professional is often characterized by an expectation of neutrality, a certain distance and a specific knowledge (Parsons 1951/2012). The encounter between the person and the professional is seldom thought of as a meeting on an equal footing (Ness 2016; Skatvedt 2017). The professionals have a certain formal power and are employed to offer specific interventions.

This special context is the basis for defining certain things as small and as “often taken for granted”, “apparently superficial”, compared to the depth of mental suffering and the scientific knowledge about these “illnesses” amassed in psychological theories. In this context small things “appear trivial” (Skatvedt 2017, p. 1).

Their importance is in their contrasting role as they “provide counter-evidence to the kind of dehumanizing treatment” many people are submitted to (Davidson and Johnson 2013, p. 259).

In other contexts than the one constituted of an unequal relationship between a professional and a person defined as in need of the professional’s help, small things are expected as part of the interactions between different persons.

### Forms

What are the different shapes that small things take? As we have already found, small things are usually things happening unnoticed in ordinary life. Thus, we might be unconscious of their very existence and of their importance to us, until they disappear from our life (Goffman 1961).

Small things take many shapes. Ness (2016) highlights that “words are not always enough (…) and that sometimes people need practical help” (59). They are also described as gestures and acts.

#### Words

Firstly, they are described as words uttered by the professional to the person.

Words are a common way to convey small things, but these words are seldom parts of complex reasoning and they might even lack a “formally therapeutic value” (Skatvedt 2017, p. 5). Small things’ words have more of a “small talk” character, “without going into details about the disease and the long-lasting diagnosis…” (Gudde et al. 2013, p. 18; Larsen and Terkelsen 2014). They might also take the form of the professional sharing experiences from his/her own life; in psychotherapeutic settings this is called “self-disclosure” (Borg and Kristiansen 2004; Costin 2016).

A small thing might consist of just one word such as a welcoming word. In other situations, it might take the form of “co-silence”, being silent together (Skatvedt 2017, p. 12).

The tone of voice is described to be just as important as the formal message the words carry (Klevan et al. 2017; Ness 2016; Costin 2016; Ware et al. 2004; Andersson 2016; Topor and Ljungberg 2016).

Finally, an aspect of words is the experience that one’s words are listened too. The specific experience of “being heard” is reported as special in many studies (Topor 2001: Ljungberg et al. 2016) as well as “being listened to” (Topor 2001; Borg and Kristiansen 2004). In the use of “Being heard” there seems to be a blurred line between a literal meaning—that the professional actually listens to and hears what is said—and a metaphorical meaning—the experience of being understood and responded to in a way that feels confirmatory (Bøe et al. 2015). “Being heard” might be the very basis for a mutual relationship, often mentioned as absent in users’ encounters with professionals (Ness 2016; Ljungberg et al. 2016).

#### Gestures

Small things described in different studies are so tiny that they can occur in public settings without been noticed, except by the persons directly engaged in the exchange. They can be described as “gestures” (Skatvedt 2017; Klevan et al. 2017), “bodily expressions” (Bøe et al. 2015) and “body language” (Costin 2016). These gestures can be produced at a spatial distance. A related concept, but one with a different spatial implication, is “bodily proximity” (Skatvedt 2017) and “presence” (Larsen and Terkelsen 2014).

The eyes seem to be an important medium for small thing; “glanses” (Skatvedt 2017) “eye contact” (Ness 2016; Ware et al. 2004) can be expressed at distance. So also can a “smile” (Skatvedt 2017; Bertelsen and Bøe 2016). On one occasion a professional cried; a bodily expression the person interpreted as: “that means that she really cared” (Bøe et al. 2015). A “hug” (Nodeland et al. 2016; Larsen and Terkelsen 2014), “the way they touched” (Andersson 2016) or “accepting a present” but also “exchange of gifts” (Borg and Kristiansen 2004; Davidson et al. 2006) often required a direct bodily contact.

Even non-gestures are mentioned, such as not answering the telephone or refusing to talk to somebody else during the meeting (Ware et al. 2004; Denhov and Topor 2012). This is interpreted as being given full attention and being the priority of the professional. This might be experienced as an “inviting attentiveness” (Bøe et al. 2015).

A common feature of gestures is their limited temporal existence. However, their symbolic effect seems to be lasting. It is also possible that their public appearance in secrecy can augment their impact, being a secret sign of complicity.

#### Actions

Actions could be defined as more extensive behaviors than gestures, often implying a specific goal and stretched over time. A characteristic of many actions mentioned was precisely their time-breaking aspect (Ljungberg et al. 2015). As for many other small things, they are ordinary actions but seem to gain a special value when they are done outside the professionals’ working hours. Actions could be sending a postcard from holidays (Denhov and Topor 2012) or an “unplanned call” for a chat (Klevan et al. 2017) or to listen to a person’s news in a difficult period (Skatvedt 2017).

A special time overrunning action occurs when a professional tells the person she/he has been thinking of him/her (Skatvedt 2017). Being invited to share a moment with the professional outside his/her professional duties is mentioned in some studies (Klevan et al. 2017; Topor 2001, Skatvedt 2017), The invitation might be just to have a smoke, take a walk or join a fishing trip. It could also include being offered “a ride, a joke, a shred of personal information, coffee and conversation about something other than mental illness, even a simple greeting” (Ware et al. 2004, p. 556; Larsen and Terkelsen 2014). It could also be about participating in the professional´s family events (Topor 2001).

“Timely responsiveness” (Ware et al. 2004) and “being available” (Borg and Kristiansen 2004) seem to play an important role for persons accustomed to being asked to wait. Another aspect of time consists of continuity, as a relationship is initiated, develops and strengthens across different common experiences (Borg and Kristiansen 2004; Denhov and Topor 2012; Topor 2014).

Taking the initiative of a shared moment, “coffee and conversation about something other than mental illness” (Ware et al. 2004, p. 556), without any hidden agenda, just for the pleasure of it contain many of the constitutive moments of small things, including the discovery of the professional as a person. As for words and silence, action might also consist of just being together, doing nothing; simply “being there” (Borg and Kristiansen 2004, p. 497).

Words, gestures, and actions seem to carry a common message from the professional to the “user”, “client”, “patient”, transforming him/her from a mere diagnosis to a person, without negating the presence of sometimes serious problems; a person that the professional (1) likes, and (2) believes has a good recovery capacity. Schrank et al. (2011, p. 234) wrote about “professional’s continued confidence in a person’s recovery”. The impact for the person of someone not giving up on them is also mentioned by Deegan (1988) and Topor (1999), even when the person him/herself has, momentarily, lost hope.

### Functions

What does small things to the person that might lead to improvements?

Two concepts present in modern recovery studies are recurrent in studies mentioning small things: mutuality and reciprocity (Slade et al. 2014; Topor 2001; Topor and Denhov 2015; Ljungberg et al. 2015; Skatvedt 2017).

In institutional contexts traditions entail a sharp division between helper and helped, and between those whose role is based on the ideas of ‘helplessness’, ‘technical incompetence’ and ‘emotional involvement’ (Parsons 1951/2012, p. 309) and those who are characterized by objectivity, neutrality and scientific knowledge’ (319) and strengthened through a range of material conditions organizing this division of power (Larsen 2009).

“To look for common ground is to emphasize the similarities between oneself and one’s service provider and to de-emphasize the differences” (Ware et al. 2004). It is about “to loosen ties rather than blindly follow the rules” (Larsen and Terkelsen 2014). A hierarchic structure is questioned (Skatvedt 2017) and the dignity of the person is re-established (Nodeland et al. 2016) in its most basic sense as, “Not stereotyped or reduced to ‘no more than’ their illness” (Ware et al. 2004, p. 557; Ljungberg et al. 2015).

It seems possible for small things to occur anywhere, but their occurrence might be favored or hindered by the characteristics of the place in which they take place. Spaces with rigorous rules that maintain a distance between staff and users and where the interactions between the two groups are open to scrutiny (wards for example, in contrast to home visits) might make it more difficult for small things to happen (Larsen and Terkelsen 2014). On the other hand, places with a social purpose might produce more occasions for small things to happen. Nevertheless, it might be true that a small thing happening in a non-permissive place might have a greater impact (Larsen and Topor 2017). But the relation between places and small things is not deterministic. It seems that also quite regulated places offer spaces and occasions where “normal” interaction between users and staff might occur (Topor 2001; Larsen 2009; Larsen and Terkelsen 2014). The existence of places for human exchange could be dependent on architectural choices. Small rooms could facilitate such sociality. A person quoted by Larsen (2009, p. 138) said: “I think it is easier to develop a more intimate contact when the rooms are not so large”.

### Consequences for the Person

Small things might be pleasurable when they occur, and might be good enough, but do they have wider consequences for the person? Some studies associated small things and their impact on hope and recovery with pleasurable experiences, and primarily unexpected ones (Schrank et al. 2011; Davidson et al. 2006).

Small things seem to have an impact on the person´s sense of self. In its most basic sense it is as small things reminded the person of him/herself as a person and not as a mere user/patient/client. Skatvedt (2017) described small things as “symbolic signs” and the interaction with the actual professional as “identity constructive encounters” (1). She also referred to Goffman´s expression of “counter-labelling” (12). Ljungberg et al. (2015) also mentioned the small things’ “symbolic value” that makes “the individual feel valued and cared for” (488).

Klevan et al. (2017) also point out that small things might be “combating the devastating effects of demoralization”.

#### Being like Others

Small things might influence the person´s sense of self primarily in conveying to him/her a sense of been just like others: “Made him feel like others”, “Ordinary” (Skatvedt 2017, 2).

Paradoxically, it seems that it is by being treated in a normal way in an un-ordinary context that the patient becomes a person that cannot be reduced to his/her diagnosis (Larsen and Terkelsen 2014). Denhov and Topor (2012, p. 4) described the following process: “... they regard the patient as an ordinary human being who is something more than merely a patient”. This is confirmed by Borg and Kristiansen (2004, p. 499), who state that small things “make the individual feel both more human and valuable, a person who means something for someone else”. “Sameness” is an adjective used by Skatvedt (2017) to describe this state.

#### As a Friend

Going beyond the boundaries of a traditional professional role reveals a need to characterize the new type of relation thus developed. One term that occurs is “as friend” (Topor 2001; Ljungberg et al. 2015; Borg and Kristiansen 2004) which stresses the paradoxical relationship, where the professional remains a professional and the user a user, but at the same time, they behave and relate to each other as friends usually do. In some studies, the relationship even evolves from “as friendship” to “friendship” (Denhov and Topor 2012). This seems to happen when the professional´s mission finishes but the relationship continues. The professional is not paid for his contact with the person and has no more formal responsibility and power in the relationship.

### Consequences for the Professional and Professionality

Small things have consequences for the persons’ sense of self. Furthermore, it seems that they might also have consequences for the professionals and for our understanding of professionality.

Small things often seem to “fall out” from the ordinariness of the contexts where they occurred and “cut across the norms” (Skatvedt 2017, p. 7).

In studies about helping professionals, the term “going beyond” has been used (Topor and Denhov 2015). What is gone beyond is “their professional role” (Borg and Kristiansen 2004, p. 501), “expertise”, “their professional responsibilities” (Laugharne et al. 2012, p. 7). Ware et al. (2004) wrote about professionals’ “willingness to go out of their way to be helpful” and in doing so they were “going above and beyond the call of duty” (556).

Terms like “breaking the rules of the institution” (Topor 2001; Borg and Kristiansen 2004) and “a breach” (Skatvedt 2017) have even be used to define how apparently trivial everyday actions could possibly have an impact on severe mental health problems.

In the process of breaking the rules and developing an “as friend” relationship it is not only the user that becomes a person; the professional also goes through the same development.

Thus, paradoxically, small things, described by users as helpful, “run the risk of being regarded as unprofessional and as representing an “irregular practice”” (Klevan et al. 2017, p. 2) and are “overlooked in studies that focus solely on illness and impairment” (Borg and Davidson 2007, p. 130).

### Consequences for practice

Small thing seems not to be the privilege of any specific profession (Denhov and Topor 2012; Costin 2016), even if “normal behaviors” might be more unexpected from professions with a higher status and thus might be expected to have a bigger impact.

#### Spontaneous

A recurring feature of small things is that they might have been done “intentionally or not” (Skatvedt 2017, p. 3; Ness 2016). In some studies, the “spontaneous” and “genuine” character of small things is stressed (Denhov and Topor 2012; Costin 2016; Ware et al. 2004). Skatvedt (2017, p. 16) wrote that they “diverge from formal protocols”.

This problematizes the vision of the planned, schedule-following and controlling professional by bringing onto the stage the possibility of emotionality and spontaneity as positive agents in a relationship between a professional and a person with mental health problems (Schrank et al. 2011).

In some studies, the importance of small things such as “self-disclosure” and other forms of “boundary crossing” is recognized, but the authors specify that these crossings should be “intentional” and “managed appropriately” (Green et al. 2008, p. 15). In these cases the clinician or professional would not be genuine but “able to determine the best approach”, thus transforming small things into appropriated technical acts based on clinical judgment of the person/patient. Here lies an important contradiction; is it possible to be genuine and spontaneous on purpose at a scheduled time and according to the patient’s diagnosis? It should be impossible to know if a small thing described as helpful was a spontaneous and genuine expression of the professional’s thoughts and feelings as a person or not (Costin 2016). It might also be a carefully planned part of the professional´s agenda. In any case it seems that a decisive aspect behind the small thing´s impact on the person is the sentiment of “genuineness” experienced by the person.

#### Unfair

Some studies stress the unfair character of small things (Topor and Denhov 2015) as they create “the sensation of receiving something special, intended for the participant alone….” (Klevan et al. 2017, p. 19). The unfairness of small things is connected to their character of “extra-ness” (Ware et al. 2004). Here it is possible to analyze two aspects of what constitute extra-ness. First, the actual small thing is beyond what the person had experienced could be expected from a staff member in the actual setting. Secondly, extra-ness appears to be something the specific professional does not do for everybody, but just for “me”; that “I” have been “singled out” (Bertelsen and Bøe 2016).

## Discussion

Our review focused on small things such as micro-affirmations, but we should keep in mind that small things might also be experienced as micro-aggressions and ways to stigmatize persons suffering from mental health problems (Gonzales et al. 2015; Peters et al. 2017). However, small things analyzed as “micro-affirmations” seem to constitute a practice helping these persons in their rebuilding of a positive sense of self and in their recovery process. Small things are part of a professional practice constituted of spontaneous signs conveying to a person with mental illness a message of shared humanity and hope.

### Small Things and Common Factors

Small things might be compared to common factors in psychotherapeutic relationships. In both cases there seems to be an emotional component, and the relation conveys hope that change is possible and that the person cannot be reduced to a diagnosis. However, there are some important differences between common factors and small things. Psychotherapies are structured interventions based on an overall hypothesis about the development of a person. Small things do not rest on a special theoretical basis. Ware et al. (2004) drew our attention to the possibility of socio-economic differences between the professional and the therapist; “…differences in role, status, and power recede into the background” (558). Rather than therapeutic alliance, they propose the notion of “social solidarity” (558). Consequently, there may be a uniqueness in small things that escapes the rationality of common factors and therapeutic alliance (Bertelsen and Bøe 2016).

### Small Things in Context

How might apparently small things have such positive impacts on mental health problems, when even the most sophisticated treatments might not? It seems to be important to place the positive small things in their context, a context dominated by belief in the efficacy of standardized, scheduled, diagnostic-based methods formulated in different guidelines. Also, a context where the importance of the relationship between person and professional is ignored or regarded as one way to obtain the person’s compliance to evidence-based methods. Without this context, small things might just be ordinary things or, as Borg and Kristiansen (2004) formulate it: “just acting in ordinary ways” (496). Nevertheless, the occurrence of an ordinary “thing” in an un-ordinary context creates the double nature of the thing, both “small” in the sense of insignificant, *and* important in its consequences.

### A Small Things Practice?

Accepting the positive consequences of small things in the reviewed studies, a conclusion could be that this knowledge about micro-affirmations *should* influence practice. However, this conclusion might be paradoxical if we keep in mind that our knowledge about small things is deeply rooted in stories about professionals’ practice. So, these micro-affirmation practices do exist and do not have to be introduced or implemented.

The problem with these apparently quite widespread and successful practices is that they are often not recognized as part of good practices in national and international guidelines. On the contrary, they are considered as counteracting the vision of offering diagnosis- and schedule-based interventions free from personal elements.

On the other hand, including small things in guidelines would transform their genuine character into established routines and obligations for the professionals. Small things are spontaneous and unfair. They seem to occur between two persons that like each other. In this sense, small things cannot be proclaimed as new rules for mental health institutions and professionals. The emotionality of professionals cannot be transformed into an intervention according to a plan. The experiences connected to small things can often be understood as a critical standpoint in relation to the actual practice in psychiatric care. Foucault (1976/1980) stressed this critical perspective when he wrote about such types of knowledges as “subjugated knowledges” (81–82); “doctors’, nurses’, psychiatric patients’ and delinquents’ knowledges”, that he defines as “unqualified, even directly disqualified knowledges” contrary to “general common-sense knowledge”, but also as so obvious that they hardly need to be specially mentioned and formulated in general rules.

## Conclusions

Knowledge about micro-affirmations and micro-aggressions could be used to create organizational conditions favorable to the emergence of micro-affirmations and improvement of the quality of the care.

In the aftermath of de-institutionalization, new ways of working have been developed. Most of these methods seem to have in common the creation of social contexts promoting the possibility for the subjectivity of the persons (users and professionals) to be expressed in social settings. Methods like “Hearing voices” (Romme and Escher 2011), “Open dialogue” (Seikkula and Olson 2003). “Individual Placement and Support” (Topor and Ljungberg 2016) and ways to organize mental health services, as in Trieste (Mezzina 2014) can be seen as conditions created to leave some space for actions, co-creations and relations for users and professionals. These methods and organizational conditions might be understood as the re-entry of normal settings into the mental health field, where the person and his/her social context are regarded as full participants in designing the help and support applied.

Research could play a role in this development by focusing on helping interventions and situations in daily practice. We should keep in mind that early studies about recovery showed a high percentage of persons in recovery (Warner 2004). The question remains: what were the helping factors behind recovery long before the age of recovery-orientated institutions and the latest bio-medical discoveries? It is also important to study micro-affirmations in everyday life, outside the professional-person relationships (Davidson et al. 2006; Topor and Ljungqvist 2017).
